# H3N2 avian influenza viruses detected in live poultry markets in China bind to human-type receptors and transmit in guinea pigs and ferrets

**DOI:** 10.1080/22221751.2019.1660590

**Published:** 2019-09-07

**Authors:** Lizheng Guan, Jianzhong Shi, Xingtian Kong, Shujie Ma, Yaping Zhang, Xin Yin, Xijun He, Liling Liu, Yasuo Suzuki, Chengjun Li, Guohua Deng, Hualan Chen

**Affiliations:** a State Key Laboratory of Veterinary Biotechnology, Harbin Veterinary Research Institute, CAAS, Harbin, People’s Republic of China; b College of Life and Health Sciences, Chubu University, Aichi, Japan

**Keywords:** Avian influenza virus, H3N2, transmission, guinea pig, ferret

## Abstract

The H3N2 influenza viruses became widespread in humans during the 1968 H3N2 pandemic and have been a major cause of influenza epidemics ever since. Different lineages of H3N2 influenza viruses are also commonly found in animals. If a different lineage of H3N2 virus jumps to humans, a human influenza pandemic could occur with devastating consequences. Here, we studied the genetics, receptor-binding properties, and replication and transmission in mammals of 15 H3N2 avian influenza viruses detected in live poultry markets in China. We found that the H3N2 avian influenza viruses are complicated reassortants with distinct replication phenotypes in mice. Five viruses replicated efficiently in mice and bound to both human-type and avian-type receptors. These viruses transmitted efficiently to direct-contact guinea pigs, and three of them also transmitted among guinea pigs and ferrets via respiratory droplets. Moreover, ferret antiserum induced by human H3N2 viruses did not react with any of the H3N2 avian influenza viruses. Our study demonstrates that the H3N2 avian influenza viruses pose a clear threat to human health and emphasizes the need for continued surveillance and evaluation of the H3N2 influenza viruses circulating in nature.

## Introduction

Influenza A viruses continue to challenge human health. The viruses are divided into different subtypes on the basis of the antigenicity of their two surface glycoproteins, hemagglutinin (HA) and neuraminidase (NA). The H1N1, H2N2, and H3N2 influenza viruses have caused four human influenza pandemics, and H1N1 and H3N2 viruses are still actively circulating in humans globally. The highly pathogenic H5 and H7 viruses often cause severe disease outbreaks in domestic poultry and wild birds. Over the last twenty years, the H5N1 viruses have not only caused damage to the poultry industries, but have also caused severe human infections and deaths in multiple countries. The H7N9 viruses that emerged in China in 2013 were low pathogenic for animals but caused severe disease in humans [1]. These viruses mutated to a highly pathogenic form in 2017 and caused influenza outbreaks in chickens in several provinces in China [2,3]. Active control strategies implemented in poultry have since essentially eliminated human infections with the H7N9 avian influenza viruses [4–6].

Low pathogenic avian influenza viruses also pose a threat to human health. The H4 and H6 avian influenza viruses are able to bind to both avian-type and human-type receptors, and some strains were able to transmit efficiently in guinea pigs via direct contact [7,8]. H9N2 viruses were transmissible in ferrets and have caused multiple human infections in several countries [9–12] . Moreover, H10 influenza viruses bearing different NA genes caused human infections in different countries [13,14]. These viruses usually do not cause disease or death in animals, which makes them low priorities for animal disease control and therefore allows them to evolve silently in nature.

The H3N2 viruses became widespread in humans during the 1968 H3N2 pandemic and have been a major cause of influenza epidemics ever since [15,16]. Of note, different lineages of H3N2 viruses are also commonly found in pigs, wild birds, and domestic poultry [17], and some avian-origin H3N2 viruses transmitted to dogs causing severe respiratory disease [18]. If a different lineage of H3N2 virus jumps to humans, a human influenza pandemic would likely occur. Here, we investigated the potential threat to public health of H3N2 avian influenza viruses by analyzing the genetics, receptor-binding properties, and replication and transmission in mammals of a series of strains that we isolated from live poultry markets in China.

## Materials and methods

### Ethics statements and facility

The present study was carried out in strict accordance with the recommendations in the Guide for the Care and Use of Laboratory Animals of the Ministry of Science and Technology of the People’s Republic of China. The protocol was approved by the Committee on the Ethics of Animal Experiments of the Harbin Veterinary Research Institute of the Chinese Academy of Agricultural Sciences.

### Virus isolation and identification

The H3N2 viruses used in this study were isolated from live poultry markets between 2009 and 2014 in China during routine surveillance. All viruses were biologically cloned three times by limiting dilution in embryonated specific-pathogen-free (SPF) eggs, and the virus stocks were grown in SPF chicken eggs and maintained at −70°C.

### Genetic and phylogenetic analyses

Virus RNA was extracted from virus-infected allantoic fluid and cDNAs were synthesized from viral RNAs by reverse transcription with Uni12 primer and amplified PCR with gene-specific primers. The complete genomes of the 15 viruses were sequenced on an Applied Bio-systems DNA analyzer. Phylogenetic analysis was performed by using the MEGA 6.0 software package, implementing the neighbor-joining method. The tree topology was evaluated by 1000 bootstrap analyses.

### Antigenic analyses

Antigenic analyses were performed by using cross hemagglutinin inhibition (HI) tests using chicken antisera generated against the selected avian viruses and ferret antisera generated against different H3N2 human viruses. We used 1.0% guinea pig red blood cells in the HI assay.

### Receptor binding analysis

Receptor binding specificity was analyzed by means of a solid-phase binding assay as described previously [7,8], using two different glycopolymers: α-2,3-sialglycopolymer [Neu5Acα2-3Galβ1-4GlcNAcβ1-pAP (para-aminophenyl)-alpha-polyglutamic acid (α-PGA)] and α-2,6-sialglycopolymer [Neu5Acα2-6Galβ1-4GlcNAcβ1-pAP (para-aminophenyl)-alpha-polyglutamic acid (α-PGA)]. Chicken antiserum against A/duck/Guangdong/S1286/09 (H3N8) virus was used as the primary antibody and a horseradish peroxidase (HRP)-conjugated goat-anti-chicken antibody (Sigma-Aldrich, St. Louis, MO) was used as the secondary antibody. Absorbance was measured at a wavelength of 490 nm.

### Studies with mice

Six-week-old female BALB/c mice (Beijing Experimental Animal Center, Beijing, China) were lightly anesthetized with CO_2_ and inoculated i.n. with 10^6^ 50% egg infective doses (EID_50_) of H3N2 avian influenza virus in a volume of 50 μL. Control mice were inoculated with 50 μL PBS. On day 3 post-inoculation (p.i.), three mice in each group were euthanized and their organs, including brains, nasal turbinates, spleens, kidneys and lungs, were collected for virus titration. The remaining five mice were monitored daily for weight loss and mortality for the following 2 weeks. The histological study of the lung sample was performed as described previously [9]. A monoclonal antibody against the NP protein of A/duck/Zhejiang/11/2000(H5N1) virus and rabbit anti-mouse IgG as the secondary antibody (Sigma-Aldrich) were used in the immunohistochemical (IHC) assays.

### Studies with guinea pigs

Hartley strain female guinea pigs weighting 300–350 g (Vital River Laboratories, Beijing, China) that were serologically negative for influenza viruses were used in our study. Ketamine (20 mg/kg of body weight) and xylazine (1 mg/kg) were used to anesthetize the animals via intramuscular injection. To investigate the replicative ability of the H3N2 viruses, groups of two guinea pigs were anesthetized and inoculated i.n. with 10^6^ EID_50_ of test virus in a 300-μl volume (150-μl per nostril). The animals were euthanized on Day 3 p.i. to collect nasal washes and lungs for virus titration. The direct contact transmission and the respiratory droplet transmission studies of the H3N2 viruses in guinea pigs were performed as previously reported [7], three pairs of animals were used for one virus. Evidence of transmission was based on detection of virus in the nasal washes and on seroconversion at the end of the 3-week observation period.

### Studies with ferrets

Four-month-old female ferrets (Wuxi Cay Ferret Farm, Jiangsu, China) that were serologically negative for influenza viruses were used in this study. The animals were anesthetized via intramuscular injection with ketamine (20 mg/kg) and xylazine (1 mg/kg). To investigate virus replication, groups of two ferrets were anesthetized and inoculated i.n. with 10^6^ EID_50_ of test virus in a 500-μl volume (250-μl per nostril). The ferrets were euthanized on day 4 p.i. and the nasal turbinates, soft palate, tonsils, trachea, lung, spleen, kidneys, and brain were collected for virus titration in eggs. For the respiratory droplet transmission studies, groups of three ferrets were inoculated i.n. with 10^6^ EID_50_ of test virus and housed in specially designed cages inside an isolator as described by Zhang et al. [1]. Twenty-four hours later, three naïve ferrets were placed in an adjacent cage (4 cm away), separated by a double-layered net divider. Nasal washes were collected from each of the virus-infected ferrets on days 2, 4, 6, and 8 p.i. and from each of the exposed ferrets on days 1, 3, 5, 7, 9, and 11 post-exposure (p.e.) and titrated in eggs. Sera were collected from all animals on day 21 p.i. or p.e. for HI antibody detection.

## Results

### The H3N2 viruses show distinct genetic diversity

**Figure 1.**
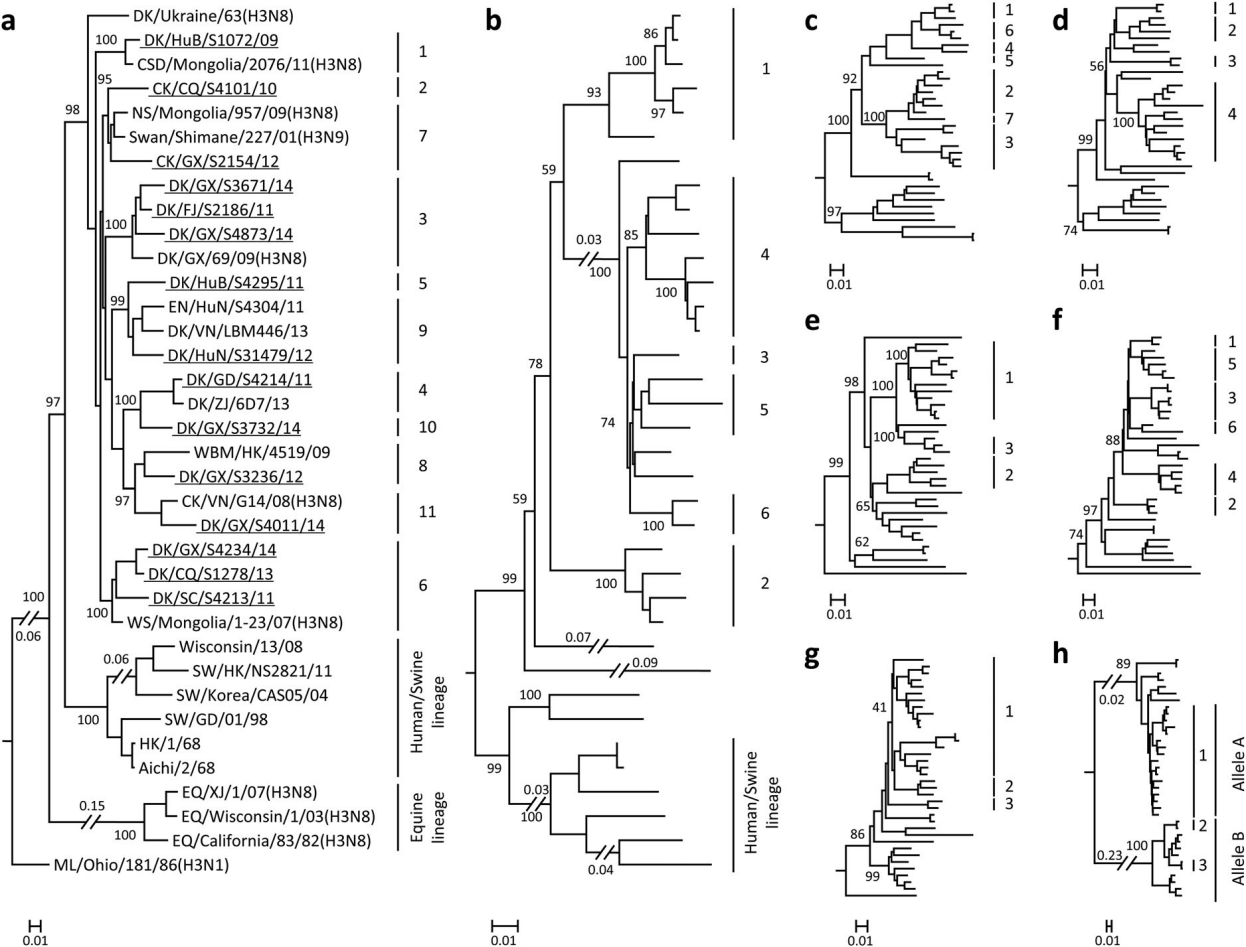
Phylogenetic analyses of H3N2 avian influenza viruses isolated from 2009 to 2014 in China. The phylogenetic trees of HA (**a**) and NA (**b**) were rooted to A/Duck/Alberta/78/1976 (H3N2) and A/Turkey/England/1969 (H3N2), respectively. The phylogenetic trees of PB2 (**c**), PB1 (**d**), PA (**e**), NP (**f**), M (**g**), and NS (**h**) were rooted to A/Equine/Prague/2/56 (H7N7). The viruses sequenced in this study are underlined in the phylogenetic tree. Abbreviations: DK, duck; CK, chicken; CSD, common shelduck; NS, northern shoveler; EN, environment; WBM, white-backed munia; WS, whooper swan; SW, swine; EQ, equine; ML, mallard; AB, aquatic bird; SBD, spot-billed duck; TK, turkey; GG, garganey; VS, velvet scoter; WWF, wild waterfowl; CT, common teal; PTD, pintail duck; GW, gadwall; RSD, ruddy shelduck; WD, wild duck; PT, pintail; MLD, mallard duck; BHG, bar-headed goose; GL, gull; MD, migratory duck; GS, goose; HuB, Hubei; CQ, Chongqing; GX, Guangxi; FJ, Fujian; HuN, Hunan; VN, Vietnam; GD, Guangdong; ZJ, Zhejiang; HK, Hong Kong; SC, Sichuan; XJ, Xinjiang; XH, Xianghai; SH, Shanghai; JS, Jiangsu; HeB, Hebei; DT, Dongting; AH, Anhui; NC, Nanchang; ST, Shantou; DG, Dongguan; WZ, Wenzhou; QH, Qinghai; JX, Jiangxi. The phylogenetic trees of Figure 1(**b–h**) with virus names are provided in Figure S1**a–g** in the supplemental material.

The 15 H3N2 viruses used in this study were isolated from apparently healthy chickens and ducks in live poultry markets during our surveillance from 2009 to 2014. The whole genome of these strains was fully sequenced. As shown in Figure 1(a), the HA genes of the 15 H3N2 avian viruses were markedly distinct from equine-, human-, and swine-origin H3 influenza viruses. The HA genes of the 15 viruses shared 87.4%–99.7% identity at the nucleotide level and formed 11 different groups in the phylogenetic tree (95% sequence identity cutoffs were used to categorize each gene group in all of the phylogenetic trees); they were closely related to the HA genes of the H3N2, H3N8, and H3N9 viruses that were previously detected in wild birds from different countries (Figure 1(a)). The NA genes of the 15 avian viruses shared 88.9%–99.6% identity at the nucleotide level and formed six groups in the phylogenetic tree, and they were closely related to the NA genes of the H5N2 and H6N2 viruses previously detected in wild birds (Figure 1(b), Figure S1a).

The six internal genes of the H3N2 viruses showed distinct diversity, with the basic polymerase 2 (PB2), basic polymerase 1 (PB1), acidic polymerase (PA), nucleoprotein (NP), matrix (M), and nonstructural protein (NS) genes of the 15 viruses sharing 87.1%–98.9%, 89.7%–99.4%, 90.0%–99.5%, 90.0%–99.7%, 92.8%–99.8%, and 71.4%–99.4% identity, respectively, at the nucleotide level. The PB2 genes formed seven groups in the phylogenetic tree (Figure 1(c), Figure S1b), and the PB1 genes formed four groups in the phylogenetic tree (Figure 1(d), Figure S1c). The PA, M, and NS genes each formed three groups in their phylogenetic trees (Figure 1(e, g and h), Figure S1d, f, and g). The NP gene formed six groups in its phylogenetic tree (Figure 1(f) and Figure S1e). Similar to the HA and NA genes, the internal genes of these viruses were derived from different strains that originated from different bird species. On the basis of this genomic diversity, we categorized the 15 viruses into 15 different genotypes (Table 1).

**Table 1.** Genotypes and replication in mice of H3N2 avian influenza viruses.

Virus	Group of each gene segment in the phylogenetic tree as shown in Figure 1								Genotype	Virus titres in organs of mice (log_10_ EID_50_) (number of virus-positive mice/total)^a^		Maximum body weight change (%)
	HA	NA	PB2	PB1	PA	NP	M	NS		Nasal turbinate	Lung	
DK/HuB/S1072/09	1	1	1	1	1	1	1	1	G1	/	3.8 ± 0.6 (3/3)	−11.8
CK/CQ/S4101/10	2	2	2	2	1	2	1	1	G2	/	/	+13.2
DK/FJ/S2186/11	3	3	3	3	2	3	1	1	G3	5.4 ± 0.3 (3/3)	4.3 ± 0.1 (3/3)	+9.1
DK/GD/S4214/11	4	4	4	4	1	4	1	1	G4	1.3 ± 0.1 (3/3)	1.4 ± 0.1 (3/3)	+11.3
DK/HuB/S4295/11	5	5	2	2	1	5	1	2	G5	/	/	+13.6
DK/SC/S4213/11	6	1	5	2	1	5	1	1	G6	/	/	+10.2
CK/GX/S2154/12	7	6	6	4	1	4	2	1	G7	1.6 ± 0.2 (2/3)	2.0 ± 0.7 (2/3)	+12.8
DK/GX/S3236/12	8	1	2	4	1	4	1	3	G8	2.5 (2/3)	2.5 (3/3)	+11.1
DK/HuN/S31479/12	9	4	2	4	1	5	1	1	G9	/	5.1 ± 0.6 (2/3)	−13.2
DK/CQ/S1278/13	6	4	7	4	1	3	1	1	G10	2.5 (1/3)	/	+8.7
DK/GX/S3671/14	3	6	2	4	2	4	1	1	G11	1.3 (1/3)	1.3 (1/3)	+11.7
DK/GX/S4873/14	3	5	3	4	3	6	2	1	G12	5.1 ± 0.7 (3/3)	3.1 ± 0.6 (3/3)	+12.0
DK/GX/S4234/14	6	4	6	4	1	3	3	1	G13	5.5 ± 0.3 (3/3)	5.2 ± 0.4 (3/3)	+9.3
DK/GX/S3732/14	10	4	3	4	2	3	1	1	G14	5.1 ± 0.6 (3/3)	5.6 ± 0.9 (3/3)	+10.1
DK/GX/S4011/14	11	4	3	4	2	3	1	1	G15	5.2 ± 0.6 (3/3)	4.5 ± 0.3 (3/3)	+8.9

^a^Groups of eight six-week-old BALB/c mice were inoculated intranasally with 10^6^ EID_50_ of each virus. Three mice from each group were killed on Day 3 post-inoculation, and virus titres were determined in samples of nasal turbinate, lung, spleen, kidney, and brain in eggs. Five mice were observed for two weeks for body weight changes. Virus was not detected from the spleen, kidney, or brain of any mouse; therefore, these data are not shown in the table. Data are presented as means ± standard deviations. /, virus was not detected in the sample.

Several amino acid changes related to the increased replication or virulence of avian influenza viruses in mammals were detected in these H3N2 viruses (Table S1). The amino acids 269S, 207K, 436Y, and 677T in PB1 [19], 383D and 515T in PA [20], and 30D and 215A in M1 [21] were conserved in all strains; the amino acids 159N in HA [22] and 42S in NS1 [23] were detected in 13 strains. The amantadine and rimantadine resistance-conferring mutation 31N in M2 was detected in one virus, whereas the amino acid 155T in HA that increases affinity for the human-type receptor appeared in all 15 strains (Table S1). None of the strains had the previously detected mammalian adaptative- and virulence-related 590S, 591K/R, 627K, or 701N residues in their PB2 gene.

### The H3N2 avian influenza viruses are antigenically similar but do not react with ferret antisera generated against human H3N2 viruses

Chicken antisera were generated against 11 H3N2 viruses that represented each of the HA groups in the phylogenetic tree. Ferret antiserum generated against six representative H3N2 human viruses that were isolated between 2007 and 2017 were also included in our HI assay. As showed in Table S2, the avian viruses cross-reacted well with antisera against the 11 avian H3N2 viruses, although the HI titres to some of the heterologous antisera were 2–4-fold lower than those to the homologous antisera. However, none of them cross-reacted with the antisera generated against the six human H3N2 viruses. The six human viruses cross-reacted well with antisera against human H3N2 viruses, although the HI titres to some of the heterologous antisera were 2- to 16-fold lower than those to the homologous antisera. Four of the six human H3N2 viruses also cross-reacted well with antisera generated against 11 chicken H3N2 viruses (Table S2). These data indicate that antisera derived from H3N2 human viruses do not cross-react with H3N2 avian viruses, although antisera derived from H3N2 avian viruses can cross-react with some H3N2 human isolates.

### The H3N2 avian influenza viruses have different replication phenotypes in mice

As shown in Table 1, three viruses, CK/CQ/S4101/10, DK/SC/S4213/11, and DK/HuB/S4295/11, were not detected in any of the organs tested; one virus, DK/CQ/S1278/13, was only detected in the nasal turbinates of mice, and two viruses, DK/HuB/S1072/09 and DK/HuN/S31479/12, were only detected in the lungs of mice; the other nine viruses were detected in both the nasal turbinates and lungs (Table 1). None of the strains were detected in the brains, spleens, or kidneys of mice (data not shown). Mice inoculated with DK/HuB/S1072/09 or DK/HuN/S31479/12 lost 11.8% or 13.2% of their body weight, respectively, whereas the mice inoculated with the other 13 viruses gained body weight (between 8.7% and 13.6%). All of the mice survived during the two-week observation period.

**Figure 2.**
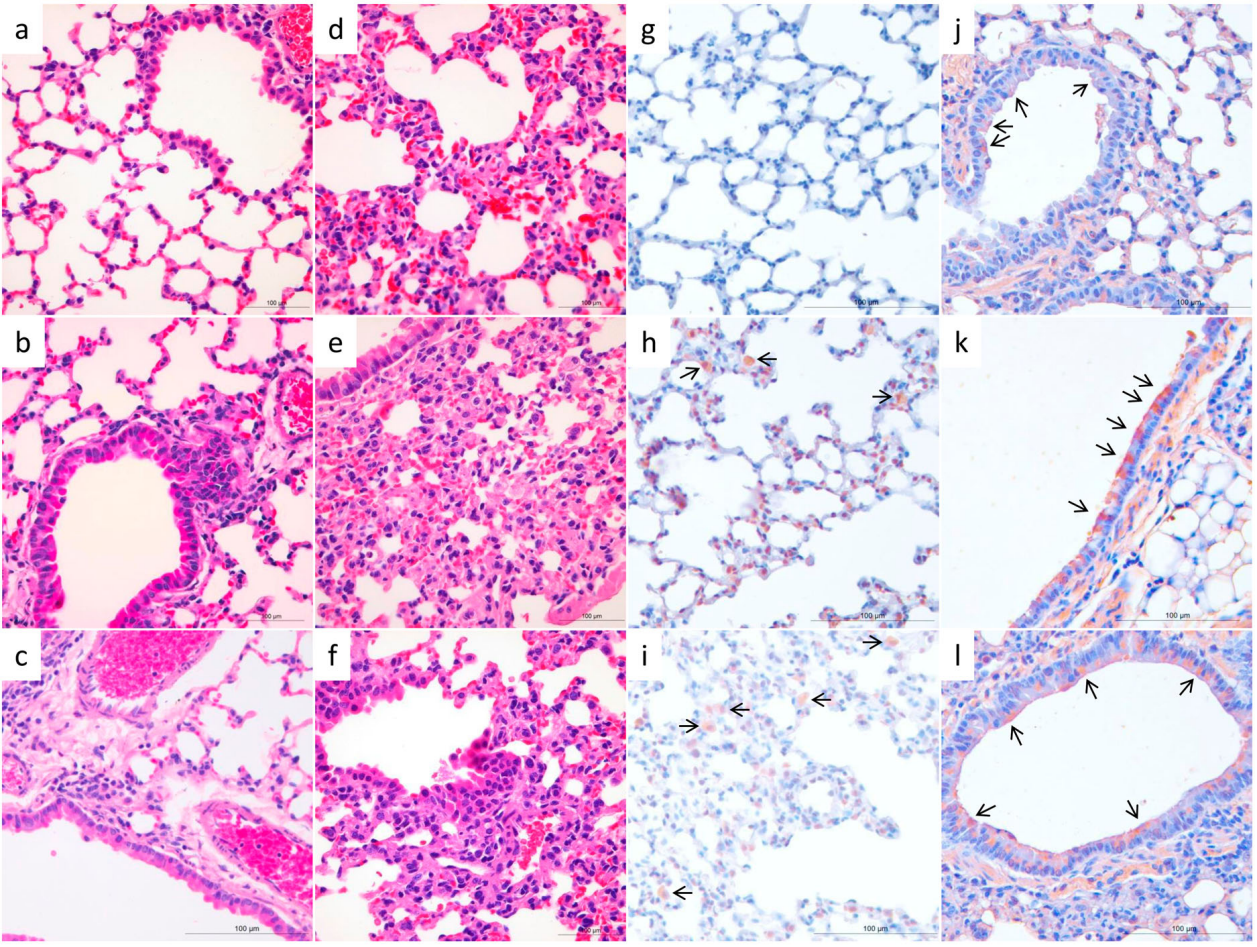
Histological study of the lungs of mice infected with H3N2 avian influenza viruses. Mice were euthanized on day 3 post-inoculation with 10^6^ EID_50_ of test virus, and the lungs were collected. **a**. The lung of PBS-inoculated control mice (H&E staining). Mild histopathological changes, including infiltration of lymphocytes near the bronchiole (**b, c**), moderate capillary congestion, hyperplasia of pneumocytes, infiltration of lymphocytes, and widening of alveolar septa were observed (**d–f**). Viral antigens were mainly detected in the alveolar macrophages and intraseptal alveolar macrophages (**h, i**), or the bronchiolar epithelial cells (**j–l**). **a–f**, H&E staining; **g–l**, immunohistochemical staining. **a** and **g**, control mice; **b** and **h**, DK/HuB/S1072/09 infected mice; **c** and **i**, DK/HuN/S31479/12-infected mice; **d** and **j**, DK/FJ/S2186/11-infected mice; **e** and **k**, DK/GX/S4011/14-infected mice; **f** and **l**, DK/GX/S4234/14-infected mice. Images were taken at 400X magnification.

We performed histological studies on lung samples from mice infected with five selected viruses. As shown in Figure 2, compared to the lung of the PBS-inoculated control mice (Figure 2(a)), mild changes were observed in the lung of virus-infected mice, including infiltration of lymphocytes near the bronchioles in DK/HuB/S1072/09- and DK/HuN/S31479/12-infected mice (Figure 2(b, c)). Similarly, we observed moderate capillary congestion, hyperplasia of pneumocytes, infiltration of a few lymphocytes, and widening of alveolar septa in the lungs of DK/FJ/S2186/11-, DK/GX/S4011/14-, and DK/GX/S4234/14-infected mice (Figure 2(d–f)). Viral antigens were mainly detected in the alveolar macrophages and intraseptal alveolar macrophages of the lung samples from the DK/HuB/S1072/09- and DK/HuN/S31479/12-infected mice (Figure 2(h, i)), or the bronchiolar epithelial cells of the lung samples from mice infected with the other three viruses (Figure 2(j–l)).

These results indicate that the H3N2 viruses circulating in avian species have the capacity to infect mice. Among 12 of the 15 viruses that were able to replicate in mice, five (DK/FJ/S2186/11, DK/GX/S4873/14, DK/GX/S4234/14, DK/GX/S4011/14, and DK/GX/S3732/14) replicated to notably higher titres than the other viruses in both the nasal turbinates and lungs of mice. We therefore selected these five viruses for receptor-binding analysis and transmissibility studies.

### The H3N2 avian influenza viruses bind to both avian-type and human-type receptors

**Figure 3.**
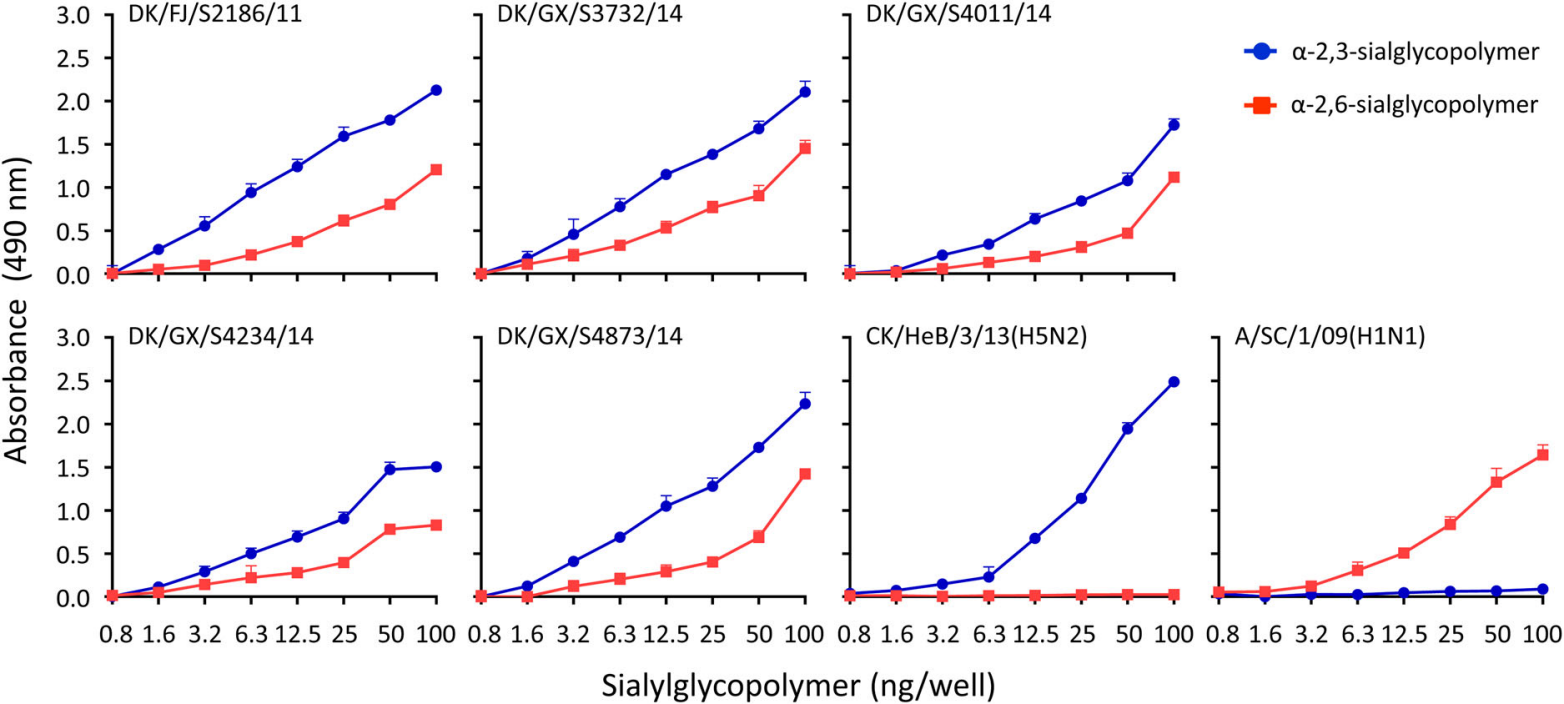
Receptor-binding properties of H3N2 avian influenza viruses. The binding of the H3N2 viruses to two different glycans (α-2,3-glycans, blue; α-2,6-glycans, red) was tested. Two viruses, CK/Hebei/3/13 (H5N2) and Sichuan/1/09 (H1N1), that bind to α-2,3- and α-2,6-glycans, respectively, were used as controls. The data shown are the means of three repeats, the error bars indicate standard deviations.

One of the critical factors governing adaptation of avian influenza viruses to humans is the glycan receptor-binding specificity of the HA protein, and the binding to α-2,6-linked sialic acids (SAs) (human-type receptor) is an essential prerequisite for influenza virus to transmit efficiently among humans [24]. We evaluated the receptor-binding properties of the five viruses that replicated efficiently in mice by using a solid-phase binding assay as reported previously [8]. We found that all of the five viruses bound to both α-2,3-linked SAs and α-2,6-linked SAs, although their affinity for α-2,6-linked SAs was lower than that for α-2,3-linked SAs (Figure 3).

### H3N2 avian influenza viruses transmit between guinea pigs

**Figure 4.**
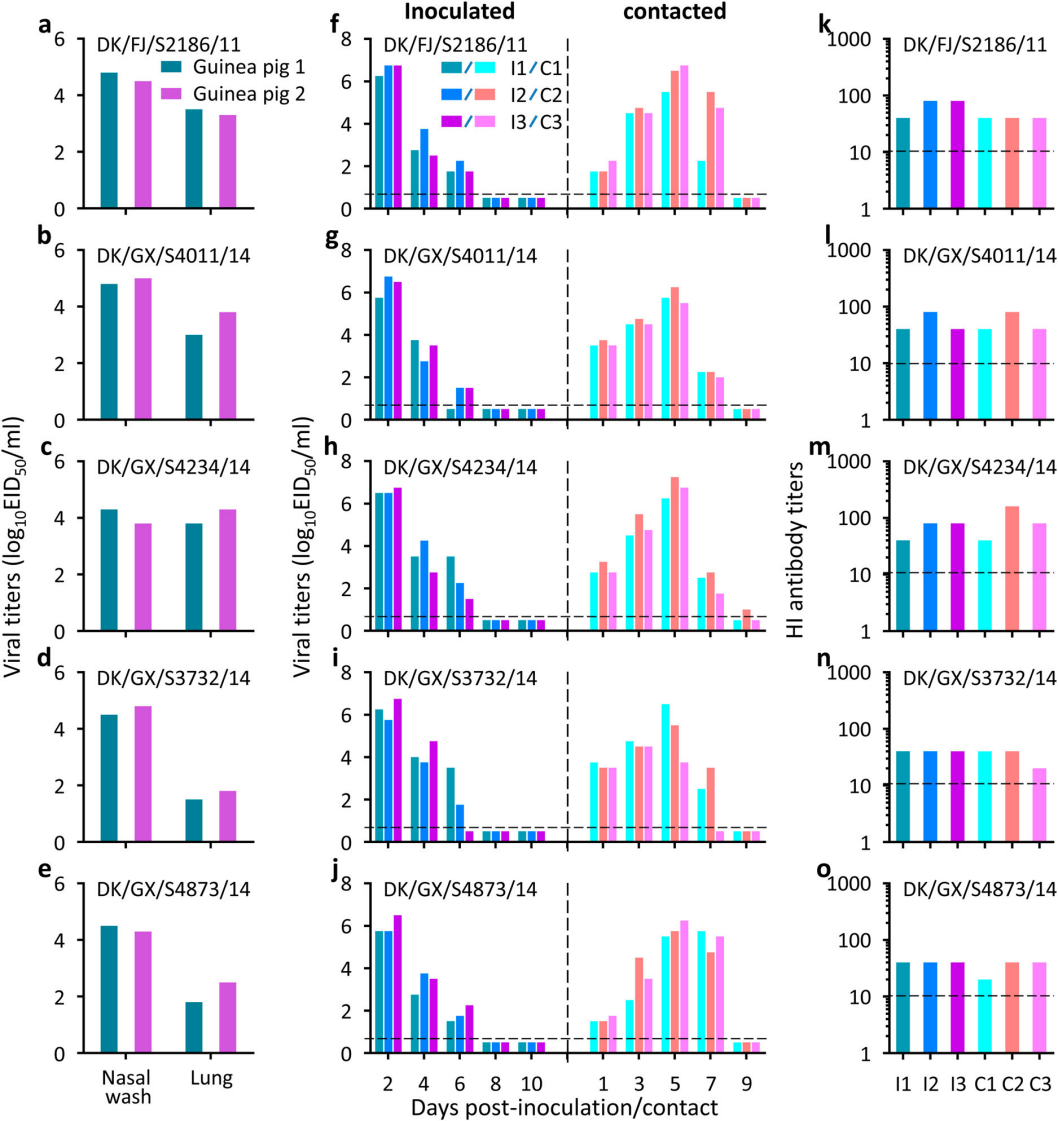
Replication and direct-contact transmission of H3N2 avian influenza viruses in guinea pigs. To test for virus replication, groups of two guinea pigs were inoculated with 10^6^ EID_50_ of test virus in a 300-μl volume (150-μl per nostril). The animals were euthanized on Day 3 post-inoculation and their nasal washes and lung were collected for virus titration in eggs. The results are shown in panels **a–e**. To test for direct contact transmission, groups of three guinea pigs were inoculated intranasally with 10^6^ EID_50_ of test virus, and 24 h later, three naïve guinea pigs were introduced into the same cage. Nasal washes were collected at the indicated times for detection of virus shedding, and seroconversion of the animals was confirmed by use of the hemagglutinin inhibition (HI) test. The virus shedding results are shown in panels **f–j**, and the HI antibody titres of the animals are shown in panels **k–o**. The horizontal dashed lines indicate the lower limit of detection. Each bar represents the virus titre or antibody titre from an individual animal.

As shown in Figure 4, three viruses, DK/FJ/S2186/11, DK/GX/S4234/14, and DK/GX/S4011/14, replicated well in guinea pigs, with titres ranging from 4.3 to 5.0 log_10_EID_50_ in the nasal washes and 3.0–4.3 log_10_EID_50_ in the lungs (Figure 4(a–c)), whereas the other two viruses, DK/GX/S4873/14 and DK/GX/S3732/14, replicated well in the upper respiratory tract with titres ranging from 4.3 to 4.8 log_10_EID_50_ but replicated relatively poorly in the lower respiratory tract with titres ranging from 1.5 to 2.5 log_10_EID_50_ (Figure 4(d and e))_._

Three pairs of guinea pigs were used to test each virus in the transmission study. We first evaluated the transmissibility of the H3N2 avian influenza viruses from inoculated guinea pigs to direct contact guinea pigs. As shown in Figure 4, virus was detected in the nasal washes of all guinea pigs that were inoculated with any of the five H3N2 avian influenza viruses, and virus was also detected in the nasal washes of the co-housed guinea pigs (Figure 4(f–j)). All virus-inoculated guinea pigs and contact guinea pigs had HI antibodies against the H3N2 viruses with titres ranging from 20 to 160 (Figure 4(k–o)). These results indicate that the H3N2 viruses can transmit efficiently from virus-inoculated animals to animals with which those animals have direct contact.

**Figure 5.**
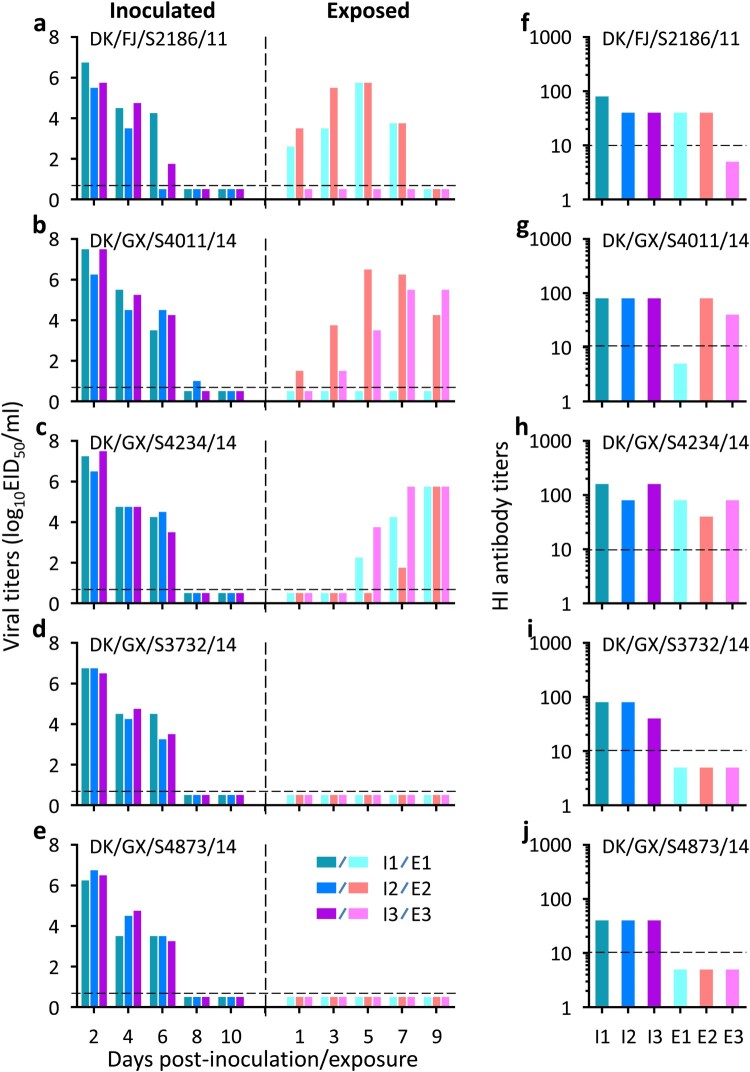
Respiratory droplet transmission of H3N2 avian influenza viruses in guinea pigs. Groups of three guinea pigs were inoculated intranasally with 10^6^ EID_50_ of test virus, and 24 h later, three naïve guinea pigs were introduced into neighbouring cages. Nasal washes were collected every 2 days from all guinea pigs beginning on Day 2 post-inoculation (p.i.) or Day 1 post-exposure (p.e.) for detection of virus shedding, and seroconversion of the animals was confirmed by use of a hemagglutinin inhibition (HI) test. Each colour bar in panels **a–e** represents the virus titre from an individual animal, and each colour bar in panels **f–j** represents the HI antibody titre from an individual animal. The horizontal dashed lines indicate the lower limit of detection.

Efficient respiratory droplet transmission from human to human is the key property required for an emerging influenza virus to cause a human influenza pandemic. We therefore evaluated the respiratory droplet transmission of the H3N2 avian influenza viruses in guinea pigs. As shown in Figure 5, virus was detected in all of the nasal washes of the virus-inoculated guinea pigs (Figure 5(a–e)). Virus was not detected in the nasal washes of guinea pigs exposed to DK/GX/S3732/14- or DK/GX/S4873/14-inoculated guinea pigs (Figure 5(d and e)). However, virus was detected in two of the three guinea pigs that were exposed to the DK/FJ/S2186/11- or DK/GX/S4011/14-inoculated guinea pigs (Figure 5(a and b)). Virus was also detected in the nasal washes of all three guinea pigs that were exposed to the DK/GX/S4234/14-inoculated guinea pigs (Figure 5(c)). Seroconversion was detected in all of the virus inoculated-animals and exposed animals that were virus-positive in terms of their nasal washes (Figure 5(f–j)). These results indicate that three of the five H3N2 avian influenza viruses tested are transmissible in guinea pigs by respiratory droplets and one of them transmits efficiently.

### Replication and transmission of H3N2 avian influenza viruses in ferrets

**Figure 6.**
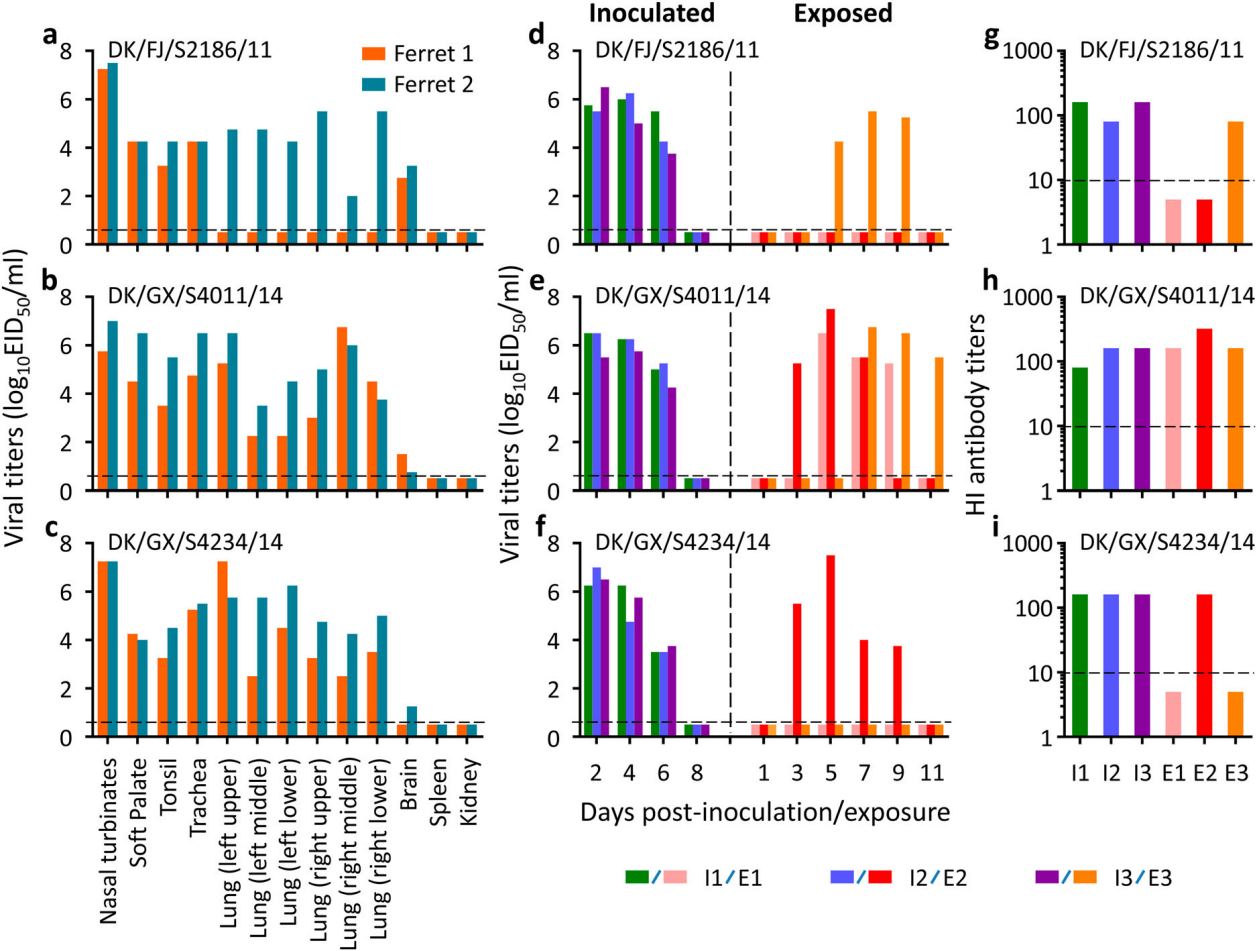
Replication and respiratory droplet transmission of H3N2 avian influenza viruses in ferrets. To test for virus replication, groups of two ferrets were inoculated with 10^6^ EID_50_ of test virus in a 300-μl volume (150-μl per nostril). The animals were euthanized on Day 4 post-inoculation and their organs as indicated were collected for virus titration in eggs. The results are shown in panels **a–c**. For the transmission study, groups of three ferrets were inoculated intranasally with 10^6^ EID_50_ of test virus, and 24-hour later, three naïve ferrets were introduced into neighbouring cages. Nasal washes were collected every 2 days from all ferrets beginning on Day 2 post-inoculation (p.i.) or Day 1 post-exposure (p.e.) for detection of virus shedding, and seroconversion of the animals was confirmed by use of a hemagglutinin inhibition (HI) test. Each bar in panels **d–f** represents the virus titre from an individual animal, and each bar in panels **g–i** represents the HI antibody titre from an individual animal. The horizontal dashed lines indicate the lower limit of detection.

As shown in Figure 6, the DK/FJ/S2186/11 virus replicated well in the nasal turbinates, soft palate, tonsils, trachea, and brain of the two ferrets inoculated, but its replication in lung was only detected in one ferret (Figure 6(a)). The DK/GX/S4011/14 and DK/GX/S4234/14 viruses were detected in the nasal turbinates, soft palate, tonsils, trachea, and lung of both inoculated ferrets. DK/GX/S4011/14 was also detected in brain of two ferrets, whereas DK/GX/S4234/14 was detected in the brain of one ferret (Figure 6(b, c)). Virus was not detected in the spleen or kidneys of any ferret.

In the transmission study, all three viruses were detected in the nasal washes of the three inoculated ferrets on days 2, 4, and 6 p.i. (Figure 6(d–f)), DK/FJ/S2186/11 and DK/GX/S4234/14 were each detected from the nasal washes of one exposed ferret, whereas DK/GX/S4011/14 was detected from the nasal washes of all three exposed ferrets (Figure 6(d–f)). All of the inoculated animals and the exposed animals that shed viruses seroconverted on day 21 p.i. or p.e. (Figure 6(g–i)). Increased body temperature was not detected from any ferrets during the observation period, but a slight decrease in body weight (≤ 8.4%) was observed in some ferrets (Figure S2). These results indicate that the three H3N2 avian influenza viruses tested are transmissible in ferrets via respiratory droplets and one of them transmits efficiently.

Previous studies have indicated that the influenza viruses can obtain certain mutations in their HA protein that may increase their affinity for human-type receptors and, therefore, their transmissibility in mammals [25,26]. For this reason, we sequenced the H3N2 viruses that we recovered from the nasal washes of ferrets in the transmission study. The G228S and both G228S and Q226R mutations of HA were respectively detected in the samples that were collected on day 6 p.i. from two of the three DK/FJ/S2186/11 virus-inoculated ferrets. This mutation was also detected in the sample that was collected from the one exposed ferret on day 9 p.e. (Table S3). The G228S, A138S, and both E190D and G228S mutations in HA were respectively detected from one of the three DK/GX/S4011/14 virus-inoculated ferrets on day 6 p.i., and the G228S mutation was detected from samples collected from all three exposed animals (Table S3). The Q226L mutation of HA was detected in the samples that were collected on day 6 p.i. from two of the three DK/GX/S4234/14 virus-inoculated ferrets, and it was also detected in the sample that was collected from the one exposed ferret on day 9 p.e. (Table S3). These results indicate that the H3N2 viruses can easily obtain mutations in their HA during replication in ferrets, although it remains to be investigated whether these viruses also obtained mutations in their other genes.

## Discussion

Here, we extensively characterized 15 H3N2 avian viruses that were isolated from live poultry markets in China, and found that H3N2 viruses circulating in avian species in nature have undergone frequent reassortment and formed complicated genotypes. The H3N2 avian viruses were antigenically similar but did not react with ferret antisera raised against representative human H3N2 viruses that were isolated between 2007 and 2017. Five of the 15 H3N2 avian viruses replicated efficiently in mice and bound to both avian-type and human-type receptors; moreover, we found that all five of these viruses transmitted efficiently to contact guinea pigs and three of them also transmitted to guinea pigs and ferrets via respiratory droplets.

Gene reassortment is an important mechanism for influenza virus evolution. Our phylogenic analysis indicated that the gene segments of the H3N2 viruses originated from different subtypes of influenza viruses previously detected in wild birds or ducks, suggesting that different influenza viruses are co-circulating and gene reassortment occurs frequently among these avian species. The fact that the 15 viruses formed 15 genotypes shows that the H3N2 viruses may not have formed a stable lineage in domestic poultry. Previous studies indicate that H9N2 influenza viruses provided their internal genes to the H7N9 and H10N8 viruses that have caused human infections in China [27,28]. Given that the H9N2 viruses are actively circulating in the live poultry markets [4,29], the H3N2 avian influenza viruses may also acquire the internal genes from the H9N2 viruses and adapt in domestic poultry or even “jump” to humans.

Replication and virulence of influenza viruses are polygenic traits. Our mouse study indicated that the H3N2 viruses have different replication phenotypes and three viruses did not replicate at all in mice, even though several amino acids in HA, PB1, PA, M1, and NS1 that confer increased replication or virulence of avian influenza viruses in mammals were present in these H3N2 avian influenza viruses (Table S1). The two viruses, DK/HuB/S1072/09 and DK/HuN/S31479/12, caused 11.8% and 13.2% mouse body weight loss, respectively, indicating that they are slightly more virulent than the other viruses tested. The immunohistochemical assays indicated that DK/HuB/S1072/09 and DK/HuN/S31479/12 mainly infected alveolar macrophages, whereas the other viruses were mainly detected in the bronchiolar epithelial cells. The different cell tropism may be related to the different virulence of these viruses in mice. Since these viruses belong to different genotypes, the precise genetic determinants that contribute to the observed biologic difference remain to be investigated.

A few amino acids in the HA protein play key roles in determining the receptor-binding properties of influenza viruses [22,30]. The amino acid 155T in HA, which has been proven to favour the binding of H9N2 virus to human-type receptors [9], was detected in all of the H3N2 avian influenza viruses in this study and also in the earliest H3N2 avian influenza virus A/turkey/England/1969 (Table S1). Therefore, HA 155T may play a key role in the binding of the H3N2 avian influenza viruses to the human-type receptors. The 226L and 228S in HA are important for the human H3N2 viruses to preferentially recognize the human-type receptors [22]. None of the H3N2 avian influenza viruses analyzed in this study had 226L or 228S in their HA; however, the Q226L mutation in HA was detected in the DK/GX/S4234/14 virus recovered from ferrets, and the G228S mutation in HA was detected in the DK/FJ/S2186/11 and DK/GX/S4011/14 viruses recovered from ferrets. In the DK/FJ/S2186/11- and DK/GX/S4234/14-exposed animals, the G228S mutation and the Q226L mutation were detected on day 9 p.e. but not earlier timepoints, indicating that these mutations may not contribute to the observed transmission of these H3N2 viruses in ferrets. The effects of these mutations on the biologic properties of avian H3N2 viruses remain to be investigated.

The live poultry markets are the major sites where humans are exposed to and become infected with different avian influenza viruses. Shi et al. isolated 619 H9N2, 273 H5, 194 H6, 191 H7, 161 H3, nine H10, and four H11 viruses from 25,471 samples that they collected in 583 live poultry markets from February 2017 to January 2018 in China, and among the 161 H3 viruses, 151 strains were H3N2 subtype^7^, indicating that the H3N2 viruses are widely circulating in live poultry markets, although they are not the most abundant subtype in these markets. Given the biologic properties of H3N2 viruses we reported here, human infection with the H3N2 avian viruses will be inevitable. Ferret antisera against different recent H3N2 human viruses did not cross-react with any of the H3N2 avian viruses in our study, which suggests that preexisting immunity may not be able to limit the spread of the H3N2 avian viruses in humans. Our study has thus revealed the risks to human health posed by H3N2 avian viruses and emphasizes the importance of continuous monitoring and evaluation of the H3N2 influenza viruses circulating in poultry.

## Supplementary Material

Supplemental Material
