# Supplementary material for: H3N2 avian influenza viruses detected in live poultry markets in China bind to human-type receptors and transmit in guinea pigs and ferrets
Source: Emerg Microbes Infect. 2019 Sep 7;8(1):1280–90. doi: 10.1080/22221751.2019.1660590 (PMC6746299; doi:10.1080/22221751.2019.1660590)

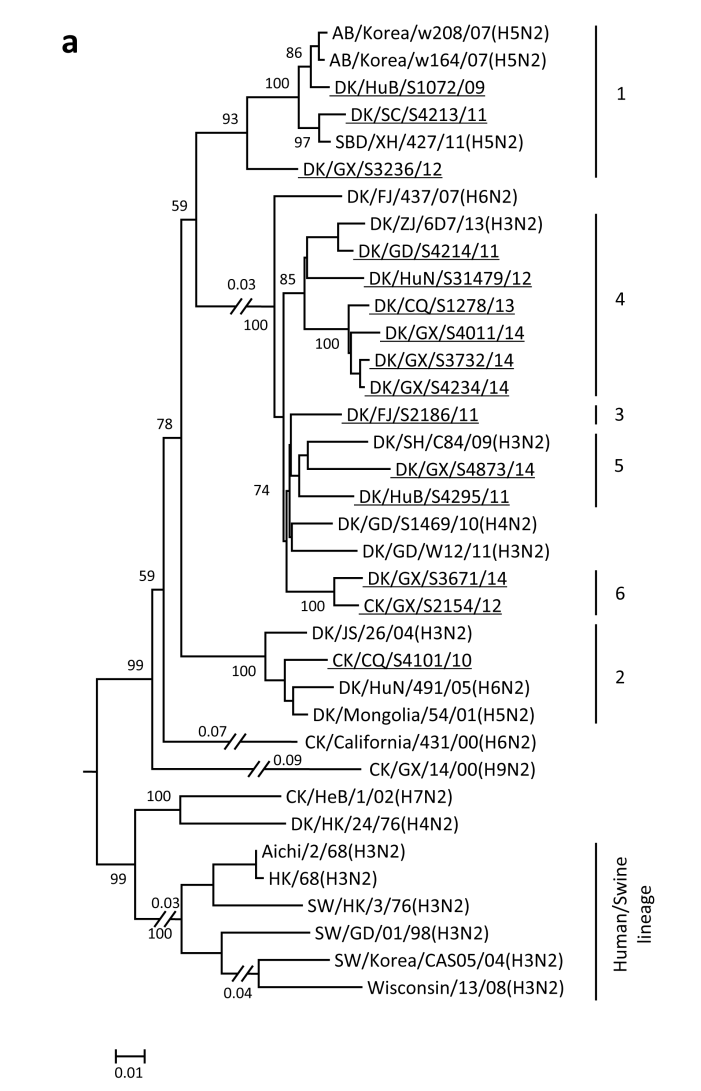


**Figure S1. Phylogenetic analyses of the NA gene and six internal genes of H3N2 avian influenza viruses isolated from live poultry markets in China.** The phylogenetic tree of NA (**a**) was rooted to A/Turkey/England/1969 (H3N2). The phylogenetic trees of PB2 (**b**), PB1 (**c**), PA (**d**), NP (**e**), M (**f**), and NS (**g**) were rooted to A/Equine/Prague/2/56 (H7N7). Abbreviations: DK, duck; CK, chicken; CSD, common shelduck; NS, northern shoveler; EN, environment; WBM, white-backed munia; WS, whooper swan; SW, swine; EQ, equine; ML, mallard; AB, aquatic bird; SBD, spot-billed duck; TK, turkey; GG, garganey; VS, velvet scoter; WWF, wild waterfowl; CT, common teal; PTD, pintail duck; GW, gadwall; RSD, ruddy shelduck; WD, wild duck; PT, pintail; MLD, mallard duck; BHG, bar-headed goose; GL, gull; MD, migratory duck; GS, goose; HuB, Hubei; CQ, Chongqing; GX, Guangxi; FJ, Fujian; HuN, Hunan; VN, Vietnam; GD, Guangdong; ZJ, Zhejiang; HK, Hong Kong; SC, Sichuan; XJ, Xinjiang; XH, Xianghai; SH, Shanghai; JS, Jiangsu; HeB, Hebei; DT, Dongting; AH, Anhui; NC, Nanchang; ST, Shantou; DG, Dongguan; WZ, Wenzhou; QH, Qinghai; JX, Jiangxi.


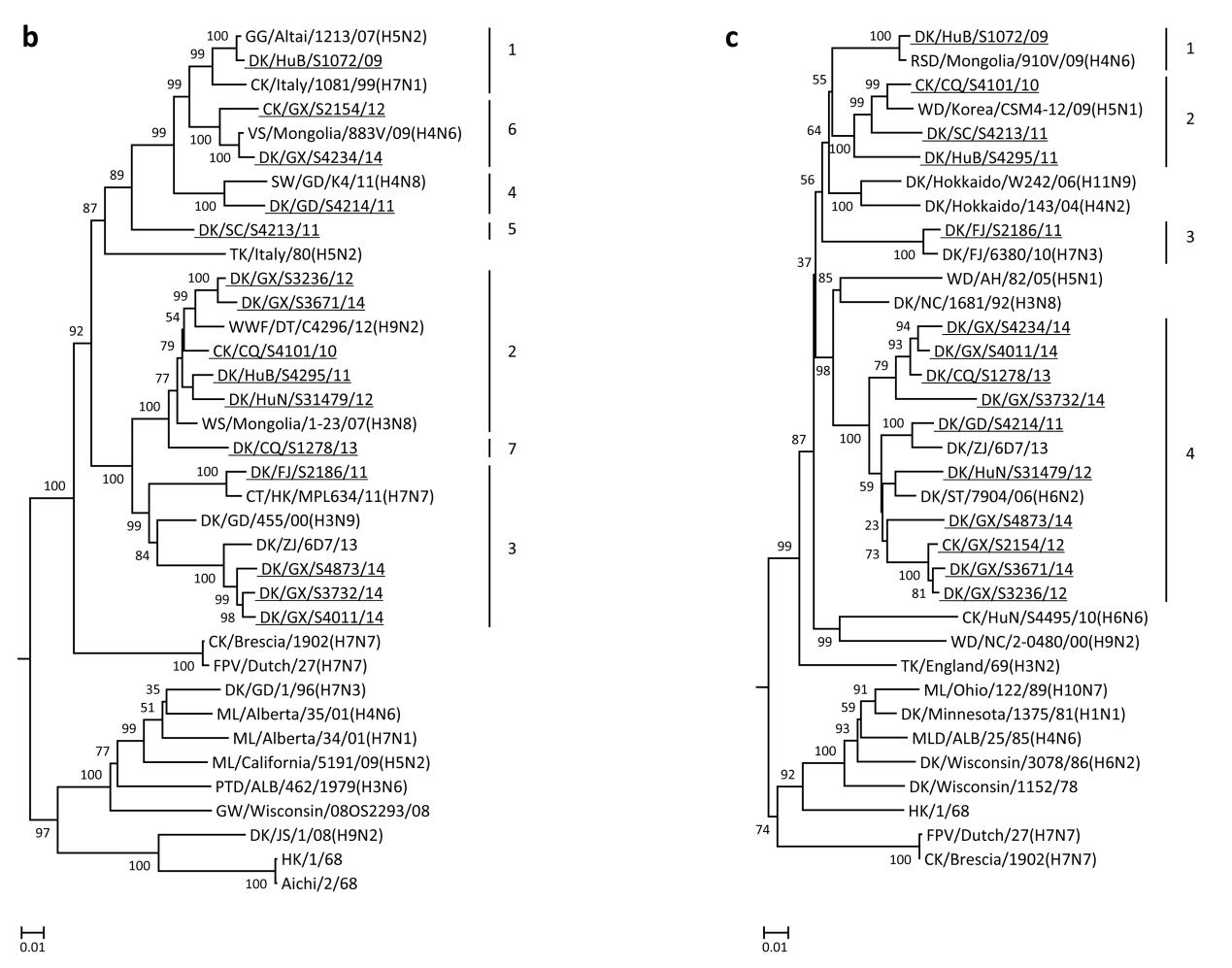


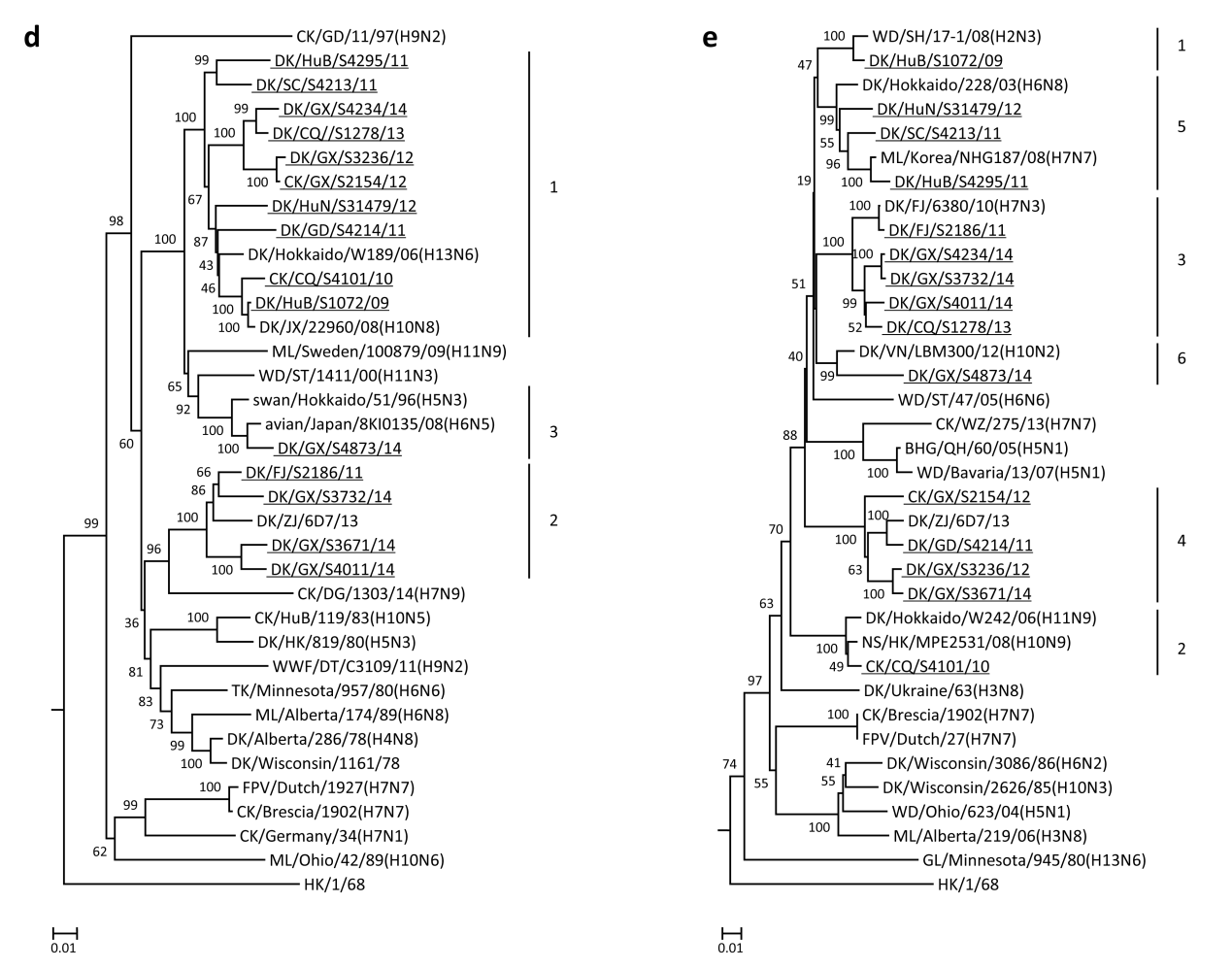


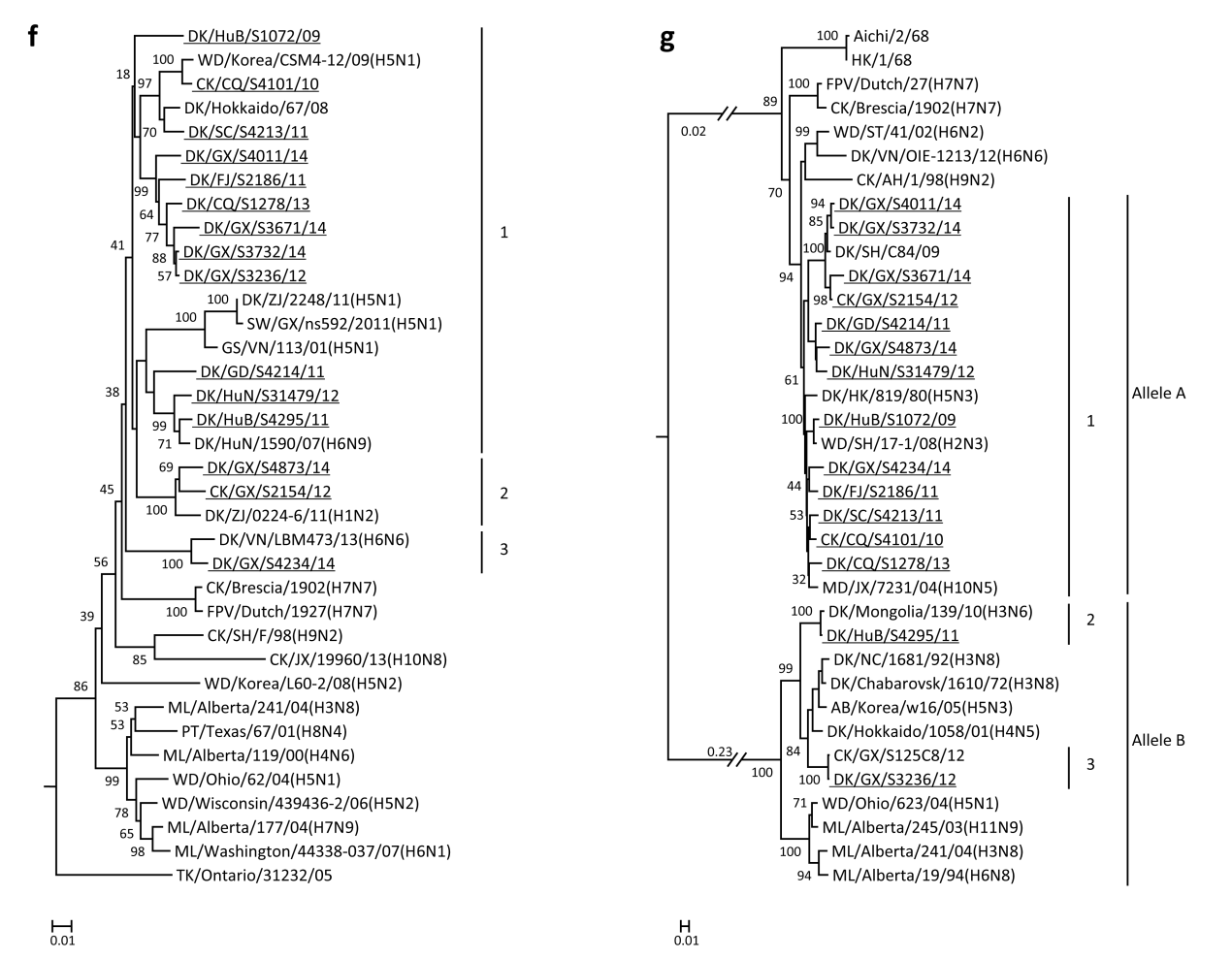

Supplement: Supplemental Material [file TEMI_A_1660590_SM1174.zip › Guan_Figure_S1_final.docx]
