# Supplementary material for: H3N2 avian influenza viruses detected in live poultry markets in China bind to human-type receptors and transmit in guinea pigs and ferrets
Source: Emerg Microbes Infect. 2019 Sep 7;8(1):1280–90. doi: 10.1080/22221751.2019.1660590 (PMC6746299; doi:10.1080/22221751.2019.1660590)
Supplement: Supplemental Material [file TEMI_A_1660590_SM1174.zip › Guan_Table_S1_final.docx]

**Table S1**. Mutations detected in the H3N2 viruses that contribute to the increased binding to human-type receptors, replication and virulence in mammals, as well as to resistance to amantadine and rimantadine.

| Virus | Mutation in HA that increases  the affinity to  human-type receptor | Mutations in different genes that increase the replication or virulence  of avian influenza viruses in mammalian hosts | | | | | | | | | | Mutation in M2 that increases the resistance to amantadine  and rimantadine |
| --- | --- | --- | --- | --- | --- | --- | --- | --- | --- | --- | --- | --- |
|  |  | HA | PB1 | | | | PA | | M1 | | NS1 |  |
|  | I155T | T159N | R207K | F269S | H436Y | M677T | N383D | A515T | N30D | T215A | P42S | S31N/D |
| A/turkey/England/1969 | T | S | K | S | Y | T | D | T | D | A | S | S |
| DK/HuB/S1072/09 | T | S | K | S | Y | T | D | T | D | A | S | S |
| CK/CQ/S4101/10 | T | N | K | S | Y | T | D | T | D | A | S | S |
| DK/FJ/S2186/11 | T | N | K | S | Y | T | D | T | D | A | S | S |
| DK/GD/S4214/11 | T | N | K | S | Y | T | D | T | D | A | A | S |
| DK/HuB/S4295/11 | T | N | K | S | Y | T | D | T | D | A | S | S |
| DK/SC/S4213/11 | T | S | K | S | Y | T | D | T | D | A | S | S |
| CK/GX/S2154/12 | T | N | K | S | Y | T | D | T | D | A | S | S |
| DK/GX/S3236/12 | T | N | K | S | Y | T | D | T | D | A | A | S |
| DK/HuN/S31479/12 | T | N | K | S | Y | T | D | T | D | A | S | S |
| DK/CQ/S1278/13 | T | N | K | S | Y | T | D | T | D | A | S | S |
| DK/GX/S3671/14 | T | N | K | S | Y | T | D | T | D | A | S | N |
| DK/GX/S4873/14 | T | N | K | S | Y | T | D | T | D | A | S | S |
| DK/GX/S4234/14 | T | N | K | S | Y | T | D | T | D | A | S | S |
| DK/GX/S3732/14 | T | N | K | S | Y | T | D | T | D | A | S | S |
| DK/GX/S4011/14 | T | N | K | S | Y | T | D | T | D | A | S | S |
