# Supplementary material for: H3N2 avian influenza viruses detected in live poultry markets in China bind to human-type receptors and transmit in guinea pigs and ferrets
Source: Emerg Microbes Infect. 2019 Sep 7;8(1):1280–90. doi: 10.1080/22221751.2019.1660590 (PMC6746299; doi:10.1080/22221751.2019.1660590)
Supplement: Supplemental Material [file TEMI_A_1660590_SM1174.zip › Guan_Table_S2_final.docx]

**Table S2. Antigenic analysis of H3N2 avian influenza viruses isolated from 2009 to 2014 in China.**

| Virus  (HA group) | Cross-reactive HI antibody titers of chicken or ferret antiserum against different H3N2 viruses^a^ | | | | | | | | | | | | | | | | |
| --- | --- | --- | --- | --- | --- | --- | --- | --- | --- | --- | --- | --- | --- | --- | --- | --- | --- |
|  | DK/HuB/S1072/09 | CK/CQ/S4101/10 | DK/FJ/S2186/11 | DK/GD/S4214/11 | DK/HuB/S4295/11 | DK/SC/S4213/11 | CK/GX/S2154/12 | DK/GX/S3236/12 | DK/HuN/S31479/12 | DK/GX/S3732/14 | DK/GX/S4011/14 | A/Brisbane/10/2007 ^b^ | A/Perth/16/2009 ^b^ | A/Texas/50/2012 ^b^ | A/Switzerland/9715293/2013 ^b^ | A/Singapore/INFIMH-16-0019/2016 ^b^ | A/Kansas/14/2017 ^b^ |
| DK/HuB/S1072/09 (1) | 256 | 256 | 64 | 128 | 128 | 256 | 256 | 128 | 256 | 256 | 256 | <10 | <10 | <10 | <10 | <10 | <10 |
| CK/CQ/S4101/10 (2) | 256 | 512 | 128 | 128 | 128 | 256 | 512 | 256 | 256 | 256 | 512 | <10 | <10 | <10 | <10 | <10 | <10 |
| DK/FJ/S2186/11 (3) | 256 | 256 | 128 | 64 | 64 | 128 | 128 | 128 | 64 | 128 | 64 | <10 | <10 | <10 | <10 | <10 | <10 |
| DK/GD/S4214/11 (4) | 64 | 256 | 64 | 256 | 128 | 256 | 256 | 128 | 256 | 256 | 256 | <10 | <10 | <10 | <10 | <10 | <10 |
| DK/HuB/S4295/11 (5) | 128 | 256 | 64 | 128 | 256 | 256 | 256 | 128 | 256 | 256 | 256 | <10 | <10 | <10 | <10 | <10 | <10 |
| DK/SC/S4213/11 (6) | 128 | 128 | 64 | 128 | 256 | 512 | 512 | 128 | 128 | 128 | 256 | <10 | <10 | <10 | <10 | <10 | <10 |
| CK/GX/S2154/12 (7) | 256 | 256 | 128 | 128 | 256 | 256 | 512 | 128 | 256 | 256 | 256 | <10 | <10 | <10 | <10 | <10 | <10 |
| DK/GX/S3236/12 (8) | 128 | 256 | 64 | 128 | 128 | 256 | 256 | 128 | 256 | 256 | 256 | <10 | <10 | <10 | <10 | <10 | <10 |
| DK/HuN/S31479/12 (9) | 128 | 256 | 128 | 128 | 64 | 128 | 128 | 64 | 64 | 64 | 64 | <10 | <10 | <10 | <10 | <10 | <10 |
| DK/GX/S3732/14 (10) | 256 | 256 | 64 | 128 | 128 | 128 | 256 | 128 | 256 | 256 | 256 | <10 | <10 | <10 | <10 | <10 | <10 |
| DK/GX/S4011/14 (11) | 128 | 256 | 64 | 128 | 128 | 128 | 256 | 128 | 256 | 256 | 256 | <10 | <10 | <10 | <10 | <10 | <10 |
| A/Brisbane/10/2007 | 32 | 32 | 32 | 32 | 32 | 64 | 32 | 32 | 64 | 64 | 32 | 640 | 40 | 320 | 640 | 80 | 160 |
| A/Perth/16/2009 | 64 | 128 | 64 | 64 | 64 | 128 | 128 | 64 | 32 | 128 | 64 | 320 | 640 | 640 | 640 | 160 | 160 |
| A/Texas/50/2012 | 64 | 128 | 64 | 64 | 64 | 128 | 128 | 128 | 128 | 64 | 32 | 640 | 80 | 640 | 640 | 40 | 640 |
| A/Switzerland/9715293/2013 | 8 | <2 | 8 | 8 | 8 | 8 | <2 | <2 | <2 | <2 | <2 | 320 | 80 | 320 | 640 | 80 | 320 |
| A/Singapore/Infimh-16-0019/2016 | 64 | 128 | 64 | 64 | 64 | 128 | 64 | 127 | 64 | 64 | 32 | 640 | 80 | 640 | 640 | 640 | 320 |
| A/Kansas/14/2017 | 8 | <2 | 8 | 8 | 8 | 16 | <2 | <2 | <2 | <2 | <2 | 320 | 640 | 640 | 640 | 320 | 640 |

^a^ Homologous titers are underlined.

^b^ The antisera against H3N2 human viruses were generated in ferrets.
