# Supplementary material for: H3N2 avian influenza viruses detected in live poultry markets in China bind to human-type receptors and transmit in guinea pigs and ferrets
Source: Emerg Microbes Infect. 2019 Sep 7;8(1):1280–90. doi: 10.1080/22221751.2019.1660590 (PMC6746299; doi:10.1080/22221751.2019.1660590)
Supplement: Supplemental Material [file TEMI_A_1660590_SM1174.zip › Guan_Table_S3_final.docx]

**Table S3.** Mutations in the HA protein of H3N2 viruses isolated in the nasal washes of ferrets in the transmission study.

| Virus | Ferret | Amino acid change(s) in HA detected at the indicated time^a^ | | | |
| --- | --- | --- | --- | --- | --- |
|  |  | Day 6 p.i. | Day 5 p.e. | Day 7 p.e. | Day 9 p.e. |
| DK/FJ/S2186/11 | Inoculated #1 | G228G&S | / | / | / |
|  | Inoculated #2 | Q226Q&R, G228G&S | / | / | / |
|  | Inoculated #3 | No mutation | / | / | / |
|  | Exposed #3 | / | No mutation | No mutation | G228G/S |
| DK/GX/S4011/14 | Inoculated #1 | G228S | / | / | / |
|  | Inoculated #2 | A138A&S | / | / | / |
|  | Inoculated #3 | E190E&G, G228S | / | / | / |
|  | Exposed #1 | / | G228S | G228S | G228S |
|  | Exposed #2 | / | G228G&S | G228G&S | / |
|  | Exposed #3 | / | / | G228S | G228S |
| DK/GX/S4234/14 | Inoculated #1 | Q226Q&L | / | / | / |
|  | Inoculated #2 | Q226L | / | / | / |
|  | Inoculated #3 | No mutation | / | / | / |
|  | Exposed #2 | / | No mutation | No mutation | Q226L |

^a^The HA genes of the viruses in the nasal washes collected from the inoculated and exposed ferrets were sequenced. Mutation was not detected in the samples collected on days 2 and 4 post-inoculation (p.i.) from the inoculated ferrets or from the samples collected on day 3 post-exposure (p.e.) from the exposed ferrets, and therefore the data from these timepoints are not shown. Amino acids are shown as single letter abbreviations and the numbers indicate the positions in the HA protein where the mutations are located. Each amino acid of the virus stock is shown before the number of the position, and each amino acid of the viruses recovered from the animals is shown after the position number. X&Y: both amino acids were detected in the sample. /, not applicable.
