# Supplementary figures and images for: H3N2 avian influenza viruses detected in live poultry markets in China bind to human-type receptors and transmit in guinea pigs and ferrets
Source: Emerg Microbes Infect. 2019 Sep 7;8(1):1280–90. doi: 10.1080/22221751.2019.1660590 (PMC6746299; doi:10.1080/22221751.2019.1660590)

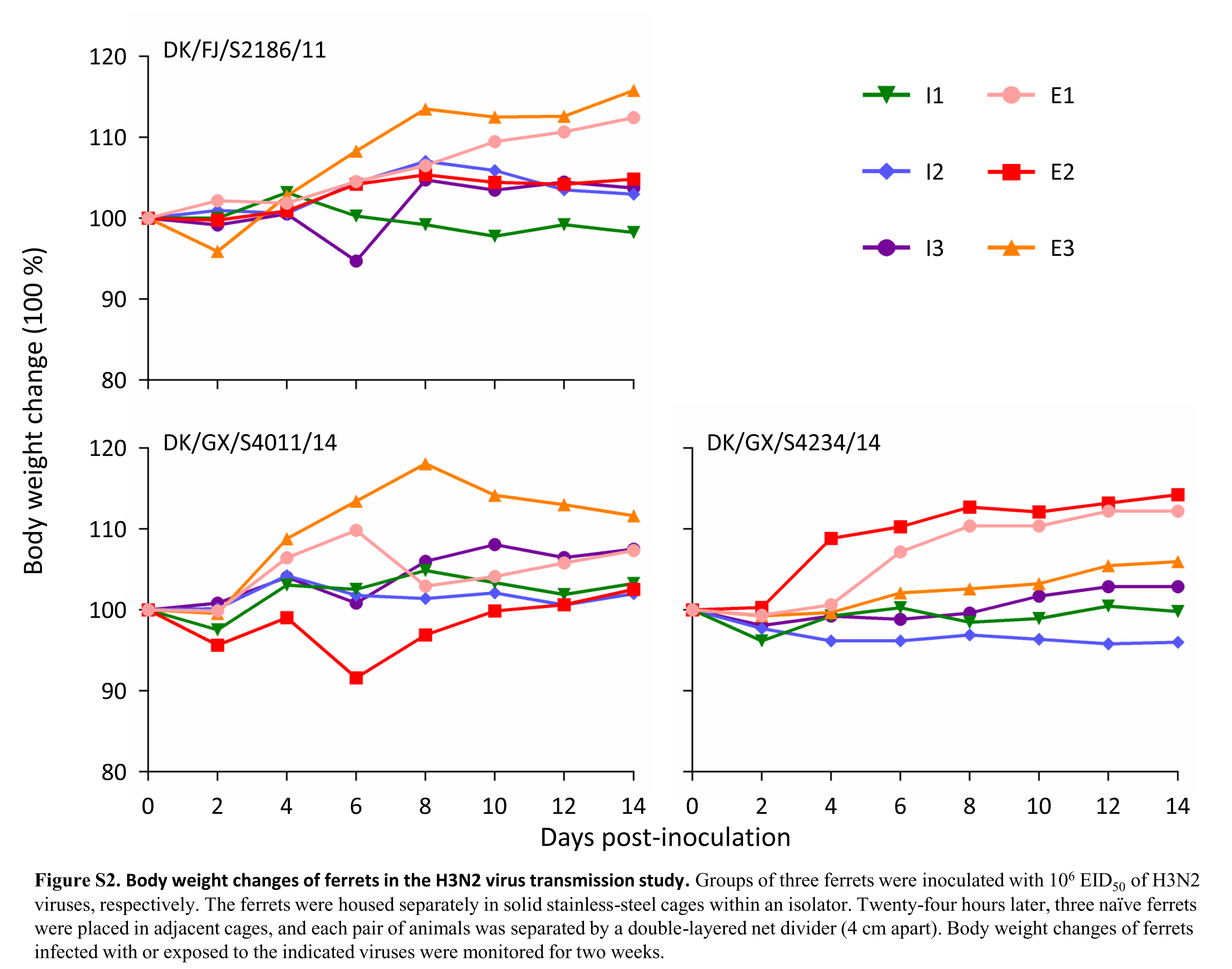

Supplement: Supplemental Material [file TEMI_A_1660590_SM1174.zip › Guan Figure S2.tif]
